# Copper Homeostasis in *Aspergillus nidulans* Involves Coordinated Transporter Function, Expression and Cellular Dynamics

**DOI:** 10.3389/fmicb.2020.555306

**Published:** 2020-11-17

**Authors:** Martzel Antsotegi-Uskola, Ane Markina-Iñarrairaegui, Unai Ugalde

**Affiliations:** Microbial Biochemistry Laboratory, Department of Applied Chemistry, Faculty of Chemistry, University of the Basque Country, San Sebastian, Spain

**Keywords:** copper toxicity response, copper uptake, *Aspergillus nidulans*, copper homeostasis, copper transporters, Ctr, di-cysteine

## Abstract

Copper ion homeostasis involves a finely tuned and complex multi-level response system. This study expands on various aspects of the system in the model filamentous fungus *Aspergillus nidulans*. An RNA-seq screen in standard growth and copper toxicity conditions revealed expression changes in key copper response elements, providing an insight into their coordinated functions. The same study allowed for the deeper characterization of the two high-affinity copper transporters: AnCtrA and AnCtrC. In mild copper deficiency conditions, the null mutant of *AnctrC* resulted in secondary level copper limitation effects, while deletion of *AnctrA* resulted in primary level copper limitation effects under extreme copper scarcity conditions. Each transporter followed a characteristic expression and cellular localization pattern. Although both proteins partially localized at the plasma membrane, AnCtrC was visible at membranes that resembled the ER, whilst a substantial pool of AnCtrA accumulated in vesicular structures resembling endosomes. Altogether, our results support the view that AnCtrC plays a major role in covering the nutritional copper requirements and AnCtrA acts as a specific transporter for extreme copper deficiency scenarios.

## Introduction

Copper (Cu) is an indispensable trace element for most living organisms. Its capacity to adopt an oxidized (Cu^2+^) and a reduced (Cu^+^) state is exploited by many enzymes to act as redox cofactor in enzyme catalyzed processes (Nevitt et al., 2012); cytochrome c, a key component of mitochondrial cellular respiration process; superoxide dismutase, for ROS neutralization; laccases, a protein family with great biotechnology implications which are involved in fungal pigment synthesis; and lysil oxidase for collagen maturation, for example (Scherer and Fischer, 2001; Lutsenko, 2010; Ding et al., 2011; Smith et al., 2017). However, free intracellular copper can interfere with red-ox processes generating reactive oxygen species (ROS) or cause metalloprotein dysfunction by displacement of other bound metal ions (Fridovich, 1983; Macomber and Imlay, 2009; Besold et al., 2016). Hence, all organisms have elaborate mechanisms that secure copper bioavailability, yet maintain free copper levels below the toxicity threshold. They include copper uptake, intracellular traffic, storage, and detoxification processes (Balamurugan and Schaffner, 2006; Nevitt et al., 2012).

Homeostasis studies in *Saccharomyces cerevisiae*, first lead to the identification of genes responsible for Cu uptake, distribution to cellular compartments and detoxification (Culotta et al., 1994; Lin et al., 1997; Pena et al., 1998). An efficient copper uptake system has been proved to be critical to cover basic cellular needs of the cation. The expression of the high affinity Cu uptake system is transcriptionally regulated by a copper metalloregulatory transcription factor (CuMRTF) named ScMac1 (Labbe et al., 1997; Keller et al., 2005). In the absence of copper, ScMac1 is able to bind DNA, thereby activating the expression of the high affinity Cu uptake system. High Cu concentrations, in turn, result in ScMac1 inactivation (Zhu et al., 1998).

Copper is transported inside the cell by two high affinity copper transporter proteins (Ctr) localized at the plasma membrane, ScCtr1 and ScCtr3 (Pena et al., 2000). They are relatively small proteins that contain up to three transmembrane domains (TM) and show high copper specificity for reduced copper (Cu^+^) (Puig et al., 2002; Petris, 2004). ScCtr1 has multiple Cu-binding methionines arranged in MxM or MxxM motifs (Mets motifs) in the extracellular N-terminal for facilitated copper import (Dancis et al., 1994). ScCtr3 on the other hand, has 11 cysteine residues, another Cu-binding ligand, throughout the sequence (Pena et al., 2000). The conserved M-xxx-M motif in the transmembrane domain is essential for copper ion translocation (Puig et al., 2002). In order to be internalized by Ctr proteins, environmental copper is reduced from Cu^2+^ to Cu^+^ by cell surface metalloreductases ScFre1 and ScFre2 prior to uptake (Rees and Thiele, 2004; Rutherford and Bird, 2004). Cu^+^ transport across membranes requires Ctr proteins to assemble as trimers and generate a pore in the plasma membrane (De Feo et al., 2009). Cu^+^ transport by Ctr proteins does not require ATP hydrolysis (Lee et al., 2002), as Cu^+^ enters the cell by a passive transport mechanism. Extracellular K^+^ and the extremely low intracellular copper concentration facilitate copper import (Balamurugan and Schaffner, 2006). The knowledge gathered on these proteins has served as a reference for further studies.

The basic structural and functional features of Ctr proteins are highly conserved in fungi and this has enabled the identification of Ctr proteins in many species. For example, in *Cryptococcus neoformans*, an opportunistic pathogen responsible for meningoencephalitis in immunocompromised individuals, high affinity Cu uptake is achieved by two Ctr proteins, denominated CnCtr1 and CnCtr4. Strains defective in copper uptake show defective melanization and reduced virulence (Ding et al., 2011; Waterman et al., 2012; Zhang et al., 2016). Melanin is a recognized virulence factor in *C. neoformans* and the laccases involved in melanin biosynthesis require Cu^+^ as a cofactor, thus copper acquisition from the host cell environment is necessary for virulence (Walton et al., 2005). Thus, besides its involvement in common cellular processes, the high affinity copper uptake has been proved to be a critical virulence factor in microbial pathogens.

For many years the study of the high affinity copper uptake system has been limited mostly to bacteria and yeast, while little was known about other organisms. However, in the last few years different manuscripts have been published in filamentous fungi. In *Aspergillus fumigatus*, a notorious opportunistic pathogen, the Ctr proteins AfCtrA2 and AfCtrC have been identified (Park et al., 2014). Studies also demonstrated that copper acquisition through the high affinity copper uptake machinery was critical for growth and conidiation in low Cu environments (Kusuya et al., 2017). As in many other pathogenic organisms, Cu uptake and virulence are also closely related in *A. fumigatus* (Cai et al., 2017). Expression of Ctr proteins is up-regulated when *A. fumigatus* conidia are challenged by human neutrophils (Sugui et al., 2008). Moreover, it has been reported that in the absence of the Ctr proteins *A. fumigatus* fails to grow in infected tissues (Cai et al., 2017), corroborating the importance of Ctr protein expression for virulence. While this research was ongoing Cai et al. (2019) published a paper describing the components of the copper uptake system in *A. nidulans*. This paper was mainly focused on the TF Mac1 and the presence of two copper transporter proteins termed, AnCtrA2 and AnCtrC, was reported.

The aim of this study was to gain insight on the effect of copper toxicity on gene expression, as well as to complement and expand the first characterization of the copper transport proteins by elucidating their role in general copper homeostasis in *Aspergillus nidulans*. For the first purpose, an RNA-seq experiment was performed which informed on gene expression changes in the most altered biological processes, structural components and molecular functions. For the second purpose, exhaustive functional analysis experiments permitted us to discern the individual roles of each Ctr protein in the copper uptake process. Apparently, AnCtrC plays a major role in high affinity copper uptake in mild copper deficiency conditions. Its deletion causes secondary level copper limitation effects like spore and pigmentation deficiencies, most likely due to Cu deficiency of the conidial laccase AnYA, which can be ascribed to limiting copper stores in the cells due to impaired copper uptake. On the other hand, AnCtrA prevails as the most effective copper uptake protein in conditions of more extreme copper deficiency. Its deletion causes primary copper limitation effects like growth limitation and lack of pigmentation. Nevertheless, individual deletion of either Ctr protein reveals that both function in a complementary manner. To further complement the characterization of the high affinity copper uptake system, a putative plasma membrane copper reductase is presented, AnFreC.

## Materials and Methods

### Bioinformatics

Alignments were performed using the predicted protein sequences released in the National Centre for Biotechnology Information (NCBI) database. Multiple sequence alignments were performed and analyzed using Clustal Omega application in EBI^1^. Transmembrane domains were predicted using Hidden Markov Models (HMM) in the Institute Pasteur Mobyle server^2^. Phylogenetic analyses were carried out using the Molecular Evolutionary Genetics Analysis version 7 (MEGA 7) software^3^.

### Strains, Media and Growth Conditions

A list of all *Aspergillus nidulans* strains used in this study is presented in Supplementary Table S1. MAD1427 (wild-type, WT) strain was used to generate single and double knock-out and GFP-tagged strains. All colonies were grown in pH 6.8 buffered solid Aspergillus minimal medium (AMM) containing Käfer’s trace elements (Kafer, 1965) 1% (w/v) D-glucose, 71 μM sodium nitrate and appropriately supplemented according to the procedure described in Pontecorvo et al. (1953). No agar was added to the medium for liquid AMM preparation. For low and high copper availability conditions, 100 μM bathocuproine disulfonic acid (BCS) Cu chelator and 100 μM CuSO_4_ were added to AMM. For the oxidative stress condition, 2-methyl-1,4-naphthoquinone (menadione) was used. AMM media prepared with Käfer’s trace elements contains 1.6 μM copper (copper-nutritional condition). For the 0 μM Cu AMM, copper was removed from the trace elements. Colony growth tests were carried out by inoculating conidiospores on solid AMM and incubating for 48 h at 37°C. Dry weight experiments were performed by inoculating conidiospores on liquid 0 μM Cu AMM, incubating for 16 h at 37°C.

Fungal growth tests were carried out in a 96-weel plate by inoculating 60 μl of a 1.10^6^ spore/ml Tween 20 0.1% suspension on 160 μl liquid AMM or AMM + 100 μM BCS and incubating for 40 h at 37°C. For BCS addition after 16 h of incubation, the required volume was added from a 12.5 μM BCS solution. Measurements are averages of three biological replicates and three technical replicates of each condition. The error bars represent the standard deviation. OD_600_ was measured with an iMark^TM^ microplate absorbance reader (Bio-Rad).

To study the effect of gene deletion on conidia production, conidiospores of mutant strains were inoculated by point inoculation in solid AMM with and without 100 μM BCS. After incubation at 37°C for 3 days, colony diameter was measured using the Digimizer image analysis software. Thereafter, in order to collect the conidia, the agar including mycelia and produced spores was vortexed in 40 μL 0.2% Tween 20 solution for 3 min. Conidia were quantified using a hemocytometer. Four biological replicates and two technical replicates of each condition/strain were performed. Two-tailed Student’s *t*-test for unpaired samples was used for the statistical analysis to compare quantitative counts of conidia (conidia/cm^2^) between the WT and mutant strains.

### Generation of Null and Tagged Strains

Deletion and C-terminally tagging cassettes were constructed following fusion-PCR technique described in Yang et al. (2004) and Markina-Inarrairaegui et al. (2011). Deletion cassettes were used for null mutant generation and contain the *Aspergillus fumigatus pyrG* or *riboB* selection marker which was amplified using the plasmid p1439 (*pyrG*^*Af*^) and p1548 (*riboB*^*Af*^) as a template with oligonucleotides gsp2^∗^ and gsp3^∗^. 1500 bp of the flanking 5′UTR and 3′UTR regions of the target gene were amplified from *A. nidulans* genomic DNA using specific primer pairs; gsp1-gsp2 (5′ UTR) and gsp3-gsp4 (3′ UTR). Hindsight, the fragments were fused using gsp1-gsp4 primers. The cassette for the generation of strains carrying C-terminally tagged fusion proteins were constructed as follow. The *gfp:riboB^*Af*^* fragment from plasmid p1548 and *mrfp:pyrG^*Af*^* fragment was amplified from plasmid p184 using oligonucleotides gsp6^∗^-gsp3^∗^. The 3′ end (∼1500 bp) of the gene and the 3′UTR regions were amplified from gDNA using oligonucleotides gsp5-gsp6 and gsp3-gsp4, respectively. Finally, oligonucleotides gsp5 and gsp4 were used to fuse the fragments. Oligonucleotides used in this study are summarized in Supplementary Table S2.

The purified fused products were then used to transform *Aspergillus nidulans* MAD1427 recipient strain following the procedure detailed in Antsotegi-Uskola et al. (2017). *pyrG*^+^ and *riboB*^+^ transformants were isolated and single integration of construct was confirmed by Southern blot technique (Supplementary Figure S1). To generate the double knock-out, strains combining a deleted allele and C-terminally tagged expressing protein strain or a double tagged strain step-by-step transformation procedure was used with individual cassettes.

The strains carrying mutations in the last cysteines of the C-terminal domain, CtrC^*C*213*A,C*214*A*^ and AnCtrA^*C*186*A,C*187*A*^ were constructed as follows: For each gene two specific complementary primers were designed, carrying the necessary nucleotide modifications for cysteine modification. Two fragments were amplified using the GFP chimera of each gene as template; first fragment was gsp5-AAreverse and the second one AAforward-gsp4. Oligonucleotides gsp5 and gsp4 were used to fuse the fragments. In the case of the C-terminally truncated mutants, other two primers were designed for each gene. The C-reverse primer was placed upstream from the last amino-acid of interest and the ^∗^C-forward primer was placed in the next nucleotide of the stop codon of the gene (5′ tail complementary to the C-reverse primer). Using the GFP chimera of each gene as a template, two fragments were amplified gsp5-C-reverse and ^∗^C-forward-gsp4. Primers gsp5 and gsp4 were used for fragment fusion. The cysteine mutants and the C-terminal deletion strains were tested by sequencing.

### RNA Isolation and RT-PCR Analysis

1.10^6^ conidia/ml were inoculated into a two liter flask with air steam containing liquid AMM and incubated at 37°C for 16 h with magnetic stirring. RNA was extracted from cells harvested prior (0 min) and after transfer to fresh liquid AMM containing 100 μM BCS or 100 μM CuSO_4_ for the indicated period. Mycelia was collected by filtration and ground in liquid nitrogen. Total RNA was isolated from one hundred milligrams of mycelia of three independent biological replicates using a RNA Isolation Kit, Nucleospin^®^ RNA plant, following instructions from the manufacturer (Macherey-Nagel GmbH & Co. KG). The integrity of the RNA was examined by gel electrophoresis and concentration was calculated using a Nanodrop 2000c system (Thermo Fisher Scientific, Waltham, MA, United States). First-strand cDNA of each sample was synthesized using the reverse transcriptase PrimeScript^TM^ RT reagent Kit (Takara) and then cDNA was used for evaluating gene expression levels. Each cDNA sample was tested in duplicate. qPCR assays were performed in an ABI 7500 system according to the manufacturer’s instructions (Applied Biosystems, Foster, CA) using 5x PyroTaq EvaGreen qPCR Mix Plus (CMB, Cultek Molecular Bioline). b*enA* was used as internal reference to normalize gene expression levels. The 2^–Δ^ ^*CT*^ method was used to determine the relative expression level ratio. Oligonucleotides used in this assay are listed in Supplementary Table S2.

### RNA-Seq

For the RNA-seq experiment 1:10 diluted supplemented AMM was used to grow MAD1427 strain. The transcriptome of vegetative hyphae grown for 16 h in liquid medium was compared with those cultured under identical conditions but shifted to 100μM CuSO_4_ medium (IC50 in 1:10 diluted media) 1 h before cell harvest. Three biological replicates were processed for each culture condition. RNA isolation, mRNA library construction, Illumina sequencing and data analysis was performed as described in Garzia et al. (2013). Briefly, total RNA was extracted based on TRIzol reagent (Invitrogen, Carlsbad, CA, United States), samples were purified using the RNeasy Mini Kit (QIAGEN, Valencia, CA, United States) and libraries were prepared following Illumina standard protocols (Illumina, San Diego, CA, United States). Sequencing was performed in a pair-end-read and 50-base mode on an Illumina HiSeq Sequencer running nine samples per lane (multiplexing). Sequences were demultiplexed and quality of reads was analyzed with FastQC v0.10.1. Reads with quality values higher than Q30 were introduced for mapping using CUTADAPT v1.2 and *Aspergillus nidulans* genome version s07-m02-r07 as the template^4^. TopHat version 2.0.6 was used for mapping reads and Cuffdiff version 2.0.2 to detect genes differentially expressed between different samples. For the determination of differentially expressed genes a *q*-value below 0.05 was used as a FDR threshold. RNA-seq results were visualized with CummeRbund version 1.99.2 software. Gene ontology (GO) terms for each *A. nidulans* gene were obtained from the *Aspergillus* genome database^5^ and were related with terms downloaded from OBO^6^. The GO project provided a standardized set of terms describing the molecular functions of genes. We used the topGO software package from the Bioconductor project^7^ to identify overrepresented GO terms from a set of differentially expressed genes. The Python programming language^8^ was used to prepare the data, utilizing rpy2^9^ to call R for the statistical analysis. The raw Illumina sequencing data were submitted to the NCBI Sequence Read Archive (SRA) and deposited under Accession Number PRJNA623550.

### Protein Isolation and Western Blot

1.10^6^ conidia were inoculated in liquid AMM and incubated on a rotary shaker at 220 rpm and 37°C for 16 h. Cells were harvested (0 min), transferred to fresh liquid AMM followed by induction with 100 μM BCS and 100 μM CuSO_4_ for the period indicated in the figures. Mycelia was collected, lyophilized for 16 h and ground in a minibeater. Protein samples were extracted by alkaline-lysis buffer (0.2 M NaOH, 0.2% β-mercaptoethanol), as described in Hervas-Aguilar and Penalva (2010).

Five micro liters of each protein sample were loaded into each well of 10% SDS-polyacrylamide gels and electrotransfered to Immun-Blot^®^ PVDF membranes by TransBlot Turbo Transfer System (Bio-Rad). Ponceau S staining was used as a loading control (0.1% Ponceau S, 5% acetic acid). Ponceau staining was removed using a 20% acetonitrile, 200 μM NaOH solution. For Western blotting the following primary antibodies were used: monoclonal mouse anti-GFP antibody (1/5000; Roche). As secondary antibodies peroxidase-conjugated goat anti-mouse IgG immunoglobulin (1/4000 dilution; Jackson Immunoresearch Lab.) is used. Proteins were detected using Clarity^TM^ Western ECL Substrate (Bio-Rad) in a Chemidoc + XRS system (Bio-Rad). Signal intensity was measured with Image Lab 3.0 software (Bio-Rad).

### Fluorescence Microscopy

For localization of AnCtrA and AnCtrC, conidiospores of GFP-tagged strains were cultured in Ibidi μ-dishes, 35 μm high (Ibidi GmbH, Germany; 2 μl of medium per well) for 16 h at 30°C in liquid Aspergillus Minimal WATCH medium (Penalva, 2005) adequately supplemented and containing 0.1% D-glucose, 71 μM sodium nitrate, 25 μM sodium phosphate monobasic. For low copper availability conditions, 100 μM bathocuproine disulfonic acid (BCS) Cu chelator were added to the WATCH media.

Fluorescence microscopy was performed using a Zeiss Axio Observer Z1 inverted microscope (63 Plan Apochromat 1.4 oil immersion Lens) and Axiocam MRm Rev.3 camera. For observation of GFP, a filter set 38 HE (Ex BP 470/40; FT 495; Em BP 525/50) was used. The images shown are representative of at least five experiments repeats. Levels of fluorescence were analyzed using open source ImageJ software^10^ (U. S. National Institutes of Health, Bethesda, MD, United States).

## Results

### Gene Expression Analysis Under Copper Toxicity Conditions

**FIGURE 1:**
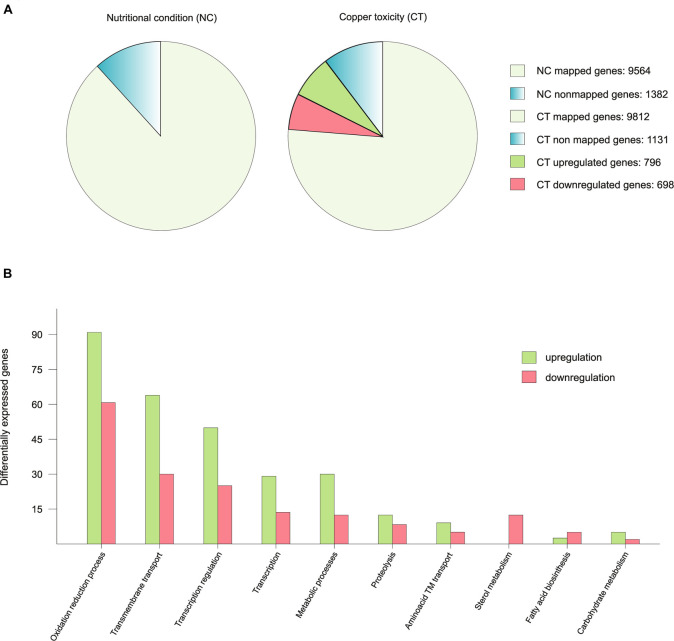
Graphic representation of the RNA-seq results. **(A)** To exemplify the number of mapped genes in each condition, the 10.943 genes predicted by the AspGD were considered as 100% and data was presented in percentages. The differentially expressed genes are represented in the copper toxicity pie chart divided in two colors in proportion to the number of up-regulated and down-regulated genes. **(B)** Representation of the up-regulated and down-regulated genes of the most affected biological processes.

An RNA-seq analysis was performed to compare the transcriptome of vegetative hyphae grown for 16 h in liquid medium (nutritional condition; acronym NC) with that of a hyphae shifted to medium with 100 μM CuSO_4_ -a high enough concentration to activate the copper toxicity response- (copper toxicity; acronym CT) 1 h before harvest. The reads of mRNAs expressed under NC and CT conditions mapped 87.4% and 89.7% of a total of 10,943 genes predicted by the AspGD, respectively. Fragments per kilobase of exon per million fragments mapped (FPKM) values for all predicted *A. nidulans* genes in each condition are shown in Supplementary Table S3. Based on the gene expression analysis criteria applied in this study (≥2-fold change, *p*-value < 0.05) the expression of 13.7% (1494) of genes was modified between the two conditions: 7.3% (796) of the genes were up-regulated (higher transcript levels in CT than in NT), while 6.4% (698) were down-regulated. The copper toxicity response therefore exhibited a balanced gene down-regulation and up-regulation rate (Figure 1A).

Table 1 shows the expression of most of the genes related to copper homeostasis, either characterized or predicted, and also some copper containing proteins. The TFs that orchestrate the copper homeostasis process, *AnaceA* and *Anmac1*, showed no significant variations in response to copper; however, the genes that code for copper mobilizing proteins, either uptake or detoxification, showed significant changes in their expression. The copper detoxification gene *AncrpA* was up-regulated in response to an external copper load. On the other hand, the already described Ctr protein coding genes *AnctrA* and *AnctrC* (elaborated on below) were significantly down-regulated under copper-toxicity condition; however, the putative Ctr protein coding gene, *AnctrB* (elaborated on below), was unaltered. By homology searches the putative homologs of the *S. cerevisiae* Atx1, Cox17 and Ccs1 have been found. A very important element for proper copper uptake is the plasma membrane oxidoreductase that reduces copper prior to internalization. In this table we show a characterized metalloreductase, *AnfreA*, and two other predicted plasma membrane oxidoreductases AN0773 and *AnfreC* (elaborated on below) that are co-regulated with the Ctr proteins and thus, could be possible metalloreductase candidates. Finally, the expression of the intracellular copper trafficking P-type ATPase *AnygA* shows a significant up-regulation under excess copper conditions.

**TABLE 1:** RNA-seq data of the copper homeostasis genes and the copper related protein coding genes.

Gene	Log2 fold change	Value_1	Value_2	*q*-Value	Significant	Description
AN1924	−0,554556	250,162	170,327	0,269576	No	*AnaceA*. Copper detoxification TF
AN0658	0,417535	20,518	27,4048	0,503204	No	*Anmac1*. Copper uptake TF
AN3117	6,4078	11,9725	1016,54	0	Yes	*AncrpA*. Copper-exporting P-type ATPase.
AN2934	−0,284851	71,0897	58,3523	0,686464	No	*AnctrB*. Copper ion transmembrane transporter activity.
AN3209	−4,18902	24,4975	1,34308	2,75E-08	Yes	*AnctrA*. High-affinity copper transporter
AN3813	−2,97333	134,667	17,1475	3,34E-12	Yes	*AnctrC*. High-affinity copper transporter.
AN3624	1,01501	20,1871	40,7964	0,015651	Yes	*AnygA*. ScCcc2 homolog. Predicted copper transporter.
AN6045	1,95069	76,4666	295,588	2,09E-07	Yes	Ccs1 SOD chaperone ortholog
AN1390	0,137135	110,504	121,523	0,853005	No	Atx1 chaperone homolog
AN4863	0,397399	78,3646	103,216	0,541532	No	Cox17 Cytochrome C oxidase chaperone homolog
AN7662	−1,11534	35,8567	16,5508	0,018818	Yes	*AnfreA*. Predicted iron metalloreductase
AN0773	−2,14409	114,6	25,9269	4,15E-08	Yes	Predicted metalloreductase
AN3208	−4,54749	14,5531	0,622335	2,56E-14	Yes	*AnfreC*. Predicted metalloreductase
AN0241	−0,342918	1361,11	1073,16	0,57373	No	*AnsodA*. Cu/Zn-superoxide dismutase
AN6635	−1,48008	4,56506	1,63643	0,034958	Yes	*AnyA*. Conidial laccase involved in green pigment production
AN6830	1,79e + 308	0	0,145851	1	No	*AnlccA*. Putative laccase
AN9170	0	0	0	1	No	*AnlccB.* Extracellular laccase
AN5379	−0,611597	0,055515	0,0363333	1	No	*AnlccC.* Extracellular laccase
AN0878	−1,92869	1,5301	0,401909	0,0384564	Yes	*AnlccD.* Extracellular laccase
AN0901	1,89527	6,9476	25,8445	6,10E-05	Yes	*AntilA*. Hyphal tip laccase

Besides copper homeostasis proteins, there are many other copper related proteins, as copper is a required cofactor of many enzymes (Table 1). The Cu/Zn superoxide dismutase *AnsodA* showed no difference in expression in response to the external copper load. On the other hand, laccases, a copper-containing enzyme-family required for melanin synthesis, displayed a divergent response: *AnyA* and *AnlccD* were down-regulated, while *AntilA* was up-regulated.

To interpret the biological significance of the registered changes in gene expression, we conducted a GO classification analysis of the results. The identified changes in gene expression occurring in response to toxic copper levels were indicative of modifications in various processes (Figure 1B). Red-ox processes were the most affected. Copper has a relatively high reduction potential (*E*^*o*^_0_ = −0.34*V*), thus, it may interfere with ongoing oxidation-reduction processes, leading to changes in the expression levels of the corresponding enzymes. For example, seven oxidoreductases with predicted roles in sterol metabolism were significantly down-regulated (AN8907, AN4094, AN6506, AN6973, AN3638, AN10648, and AN3817). Catalases *AncatB*, *AncatC* and *AncpeA*/*AncatD* were slightly up-regulated. On the other hand, the expression of superoxide dismutases and the oxidative stress transcription factors *AnnapA* and *AnatfA* was not altered. Transmembrane transport of various substrates, as sugars, oligopeptides and metals was modified: the xylose transporters *AnxtrA* and *AnxtrB*, the putative high affinity nickel uptake protein AN6115, the putative Cation Diffusion Facilitator (CDF) transporter. Finally, transcriptional regulation was also notably altered, based on the changes in the expression of transcription factors. Most of them were unidentified.

Analyzing the repercussion of copper-toxicity on gene expression we found that certain gene clusters were affected by copper addition. For example, the siderophore biosynthesis gene cluster (*AnsidA*, *AnsidC, AnsidD, AnsidF, AnsidG, AnsidH*, and *AnsidI*) was notably down-regulated, together with the iron homeostasis TF, *AnhapX*. Besides the siderophore biosynthesis cluster, most members of the Emericellamide antibiotic biosynthesis gene cluster were down-regulated (*AneasB*, *AneasC*, and *AneasD*). A set of genes (AN2558, AN2559, AN2560, AN2561, and AN2562) with no confirmed function was drastically upregulated. However, by sequence analysis tools we found that AN2562 is the homolog of the *N*-acetylglucosamine transporter Ngt1 from *C. albicans;* AN2560 and is the homolog of the high-affinity methionine and cysteine transporter Mup1 from *S. cerevisiae* and *C. albicans*; AN2558 is the homolog of the flavin-containing monooxygenases CaIfk1 and ScFmo1; and AN2559 and AN2561 are a predicted oxidoreductase and a predicted methyltransferase, respectively (Table 2).

**TABLE 2:** RNA-seq data of differentially expressed genes.

Gene	Log2 fold change	NC	CT	*q*-Value	Significant	Description
AN8251	−2,29289	212,998	43,4658	2,21E-10	Yes	*AnhapX*. TF iron homeostasis
AN5823	−1,6464	2071,32	661,656	0,00046089	Yes	*AnsidA*. Siderophore biosynthetic gene cluster member
AN8751	0,252704	33,0097	39,329	0,68274	No	*AnsidB*. Siderophore biosynthetic gene cluster member
AN0607	−0,957512	17,4648	8,99339	0,0248114	Yes	*AnsidC*. Siderophore biosynthetic gene cluster member
AN6236	−1,68316	454,38	141,494	0,00026402	Yes	*AnsidD*. Siderophore biosynthetic gene cluster member
AN6234	−0,91275	248,15	131,81	0,0336505	Yes	*AnsidF*. Siderophore biosynthetic gene cluster member
AN8539	−1,19743	277,515	121,011	0,00473987	Yes	*AnsidG*. Siderophore biosynthetic gene cluster member
AN6235	−1,19947	157,372	68,5252	0,00438479	Yes	*AnsidH*. Siderophore biosynthetic gene cluster member
AN0609	−2,13608	394,676	89,7878	3,63E-09	Yes	*AnsidI*. Siderophore biosynthetic gene cluster member
AN10080	−0,216896	22,965	19,7594	0,777446	No	*AnsidL*. Siderophore biosynthetic gene cluster member
AN7800	−0,267815	48,5357	40,3126	0,702964	No	*AnmirA*. Siderophore iron transporter
AN8540	−1,24341	1759,6	743,208	0,0167845	Yes	*AnmirB*. Siderophore iron transporter
AN7485	−0,738315	101,585	60,894	0,103354	No	*AnmirC*. Siderophore iron transporter
AN8907	−3,85352	2257	156,138	0	Yes	Putative sterol methyl oxidase
AN4094	−2,81214	478,501	68,131	2,56E-14	Yes	Putative sterol reductase
AN6506	−2,63572	947,541	152,464	2,10E-11	Yes	Putative sterol methyl oxidase
AN6973	−1,72893	1740,29	525,004	5,21E-05	Yes	Putative sterol methyl oxidase
AN3638	−1,34834	382,258	150,129	0,00162595	Yes	Putative sterol methyl oxidase
AN10648	−0,960416	76,6647	39,3987	0,0282487	Yes	Sterol reductase activity, ergosterol biosynthetic process
AN3817	−0,929126	85,5385	44,9228	0,0288343	Yes	Putative reductase, predicted role in sterol metabolism
AN6412	1,75949	4,36251	14,7705	0,00418067	Yes	*AnxtrA.* Xylose transporter A
AN3264	1,44359	4,83779	13,1587	0,0126647	Yes	*AnxtrB.* Xylose transporter B
AN2911	−0,0516925	117,662	113,521	0,95033	No	*AnatfA.* TF for response of conidia to stress.
AN7513	0,53883	117,309	170,426	0,298838	No	*AnnapA*. TF required for resistance to oxidative stress
AN8637	−1,56595	4,86518	1,64324	0,0317285	Yes	*AncatA*. Catalase
AN9339	1,49778	18,7857	53,0525	0,00017666	Yes	*AncatB*. Catalase
AN5918	1,24056	25,0035	59,0807	0,00396133	Yes	*AncatC*. Catalase
AN7388	1,37382	12,0197	31,1495	0,00400481	Yes	*AncpeA/AncatD*. Catalase
AN6115	1,89337	35,271	131,032	1,45E-06	Yes	Cation Diffusion Facilitator (CDF) transporter
AN2545	−1,46337	0,478858	0,173655	1	No	*AneasA*. Emericellamide biosynthetic gene cluster
AN2547	−2,25667	1,10672	0,231587	0,001229	Yes	*AneasB*. Emericellamide biosynthetic gene cluster
AN2548	−4,52491	0,952086	0,0413563	0,0229756	Yes	*AneasC*. Emericellamide (eas) biosynthetic gene cluster
AN2549	−2,98507	14,804	1,86976	1,06E-07	Yes	*AneasD*. Emericellamide (eas) biosynthetic gene cluster
AN2558	6,95672	0,0388284	4,82314	5,62E-05	Yes	CaIfk2 and ScFmo1 homolog
AN2559	8,11636	0,171753	47,662	2,56E-14	Yes	Predicted oxidoreductase activity
AN2560	7,69094	0,0580346	11,992	6,81E-07	Yes	ScMup1 and CaMup1 homolog
AN2561	7,98542	1,19049	301,7	0	Yes	Putative methyltransferase
AN2562	7,07374	0,0340531	4,58737	0,00125881	Yes	CaNgt1 homolog

In summary, copper toxicity altered the expression of 13.7% of the genes with a balanced ratio between up-regulated and down-regulated genes (Figure 1A). Changes in copper concentration derived in a strong modification of the copper homeostasis system, the copper dependent enzymes and multiple biological processes, especially oxidation reduction processes and predictably, in cellular components such as biological membrane composition. As a remarkable feature, in most of these processes, more genes were up-regulated than down-regulated (Figure 1B).

### Screening for Potential Copper Transport Proteins

In order to select putative copper transporters we searched for putative orthologs of *Saccharomyces cerevisiae* Ctr transporters among down-regulated genes under copper toxicity. Blastp searches using the full-length of the ScCtr protein sequences yielded three results, the already identified AN3209 and AN3813, and another putative copper transporter, AN2934, termed *AnctrB*. *AnctrB* is the top hit of the low affinity copper transporter ScCtr2, which encodes a vacuolar Cu transporter (Liu et al., 2012; Qi et al., 2012). Cai et al., 2019 recently identified AN3209 and AN3813 as copper uptake proteins in *A. nidulans*, and AnMac1 as the TF controlling their expression. In this work, AN3209 and AN3813 were named AnCtrA2 and AnCtrC, respectively, claiming they were AfCtrA2 and AfCtrC homologs, but the protein sequence analysis results support a different interpretation. BLASTp analyses revealed that AnCtrA and AnCtrC are significant hits of AfCtrC (42% and 60% protein identity, respectively); however, there is no significant hit of AfCtrA2 (24% and 22% protein identity, respectively) in the *A. nidulans* proteome. Thus, instead of naming AN3209 as AnCtrA2, we considered the name AnCtrA.

Sequence data revealed that *AnctrA* and *AnctrC* sharegreat similarity. Furthermore, out of the 127 species that possess an *AnctrA* ortholog, 94 also possess an *AnctrC* ortholog (Figure 8).

**FIGURE 2:**
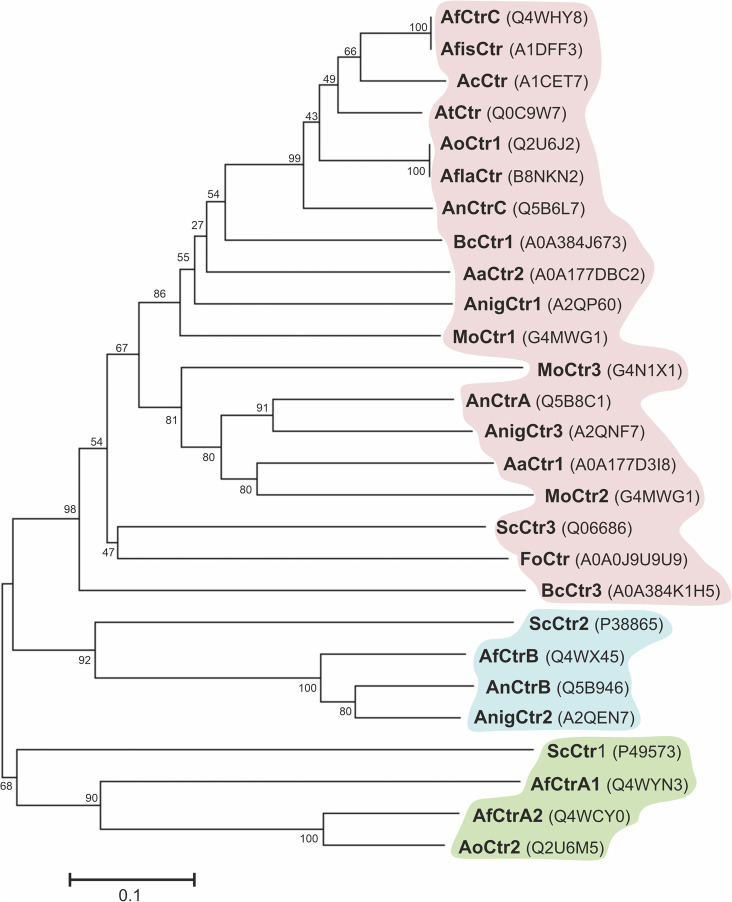
Phylogenetic analysis of Ctr proteins. Using MEGA7, a phylogenetic tree was generated by the Neighbor-Joining method with the Bootstrap method as phylogeny test. Analyzed organisms: *Alternaria alternata* (Aa), *Aspergillus nidulans* (An), *Aspergillus fumigatus* (Af), *Aspergillus fischeri* (Afis), *Aspergillus flavus* (Afla), *Aspergillus niger* (Anig), *Aspergillus oryzae* (Ao) *Aspergillus clavatus* (Ac), *Aspergillus terreus* (At), *Botrytis cinerea* (Bc), *Fusarium oxysporum* (Fo), *Magnaporthe oryzae* (Mo), and *Saccharomyces cerevisiae* (Sc).

According to the phylogeny analyses shown in Figure 2, three major clusters can be differentiated. Most Ctr proteins of filamentous fungi are related to ScCtr3 (red); *A. fumigatus*, *A. nidulans*, and *A. niger* possess Ctr proteins closely related to the vacuole Cu transporter ScCtr2 (Blue); Out of the 27 proteins of the phylogeny test, only four proteins are related to ScCtr1 (Green). The results reflect that ScCtr3 orthologs comprise the biggest group of proteins and in this group we can find most Ctr proteins of filamentous fungi.

**FIGURE 3:**
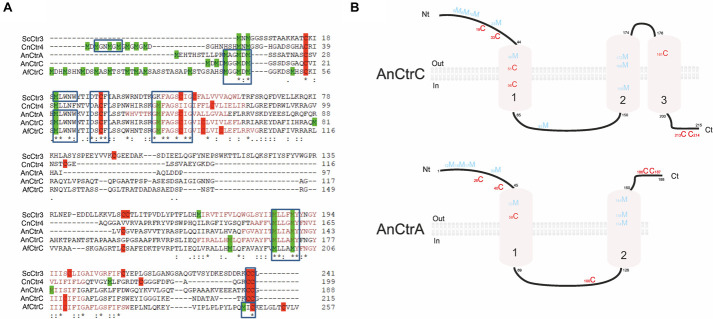
Sequence analysis of *Aspergillus nidulans* high affinity copper transporters AnCtrA and AnCtrC. **(A)** Alignment of predicted full-length of *Saccharomyces cerevisiae* Ctr3, *Cryptococcus neoformans* Ctr4, *Aspergillus nidulans* CtrA and CtrC, and *Aspergillus fumigatus* CtrC sequences compared using the Clustal method. Identical residues are described with asterisk, and conservative and semi-conservative residues with double and single dots, respectively. Methionine residues are represented in green and cysteines in red. The length of each protein is indicated adjacent to the last amino acid. Protein accession numbers are reported as follows: ScCtr3 (Q06686), CnCtr4 (J9VLN4), AnCtrA (C8VIA1), AnCtrC (C8V6S8), and AfCtrC (Q4WHY8). **(B)** Proposed model of AnCtrA and AnCtrC describing the predicted membrane topology and the conserved methionine and cysteine residues. AnCtrA contains two transmembrane domains and AnCtrC three.

A multiple sequence alignment of *A. nidulans* Ctr proteins is shown in Figure 3A. Strong similarities in amino acid sequences could be observed, except for the loop between transmembrane domains (TMD) 1 and 2, which showed higher variability. Detailed sequence analysis showed the presence of common conserved motifs predicted to code for copper binding. AnCtrA, AnCtrC, AfCtrC and CnCtr4 contain a Met motif arranged as M-xx-M-x-M in the amino-terminal region (Nt) (Met motif shown in blue box). Three additional methionine residues are conserved among the Ctr protein sequences aligned: a methionine in the amino-terminal, located ∼ 22 amino acids upstream from the TMD1 (M^30^ in AnCtrA, M^23^ in AnCtrC, M^20^ in ScCtr3, M^58^ in AfCtrC and M^41^ CnCtr4) and an M-xxx-M motif in the predicted TMD2 (M^134^-M^138^ in AnCtrA, M^168^-M^172^ in AnCtrC and M^185^-M^189^ in ScCtr3, M^197^-M^201^ in AfCtrC and M^156^-M^160^ CnCtr4). The methionine residue located prior to TMD1 is equidistant in *A. nidulans* and other fungi, as noted by Puig et al. (2002). As shown in Figure 3A, there are two conserved cysteine residues flanking this functional residue (Cys residues are highlighted in red for their copper handling capacity). Additional methionine and cysteine residues are widespread within the sequence. Finally, in all the proteins analyzed, close to the terminus of the carboxy-terminal region a putative copper handling motif that could be involved in copper coordination and transport was observed; a di-cystine motif in the case of ScCtr3, CnCtr4, AnCtrA and AnCtrC, and a M-x-C motif in the case of AfCtrC. Based on computer algorithms (TMHMM and HMMTOP predictions), as shown in Figure 3B, AnCtrA was predicted to contain two transmembrane regions while AnCtrC contained three. According to these predictions, the amino tail would be oriented to the outside of the cell and the carboxyl tail into the cytosol, in the case of AnCtrC. In the case of AnCtrA, however, both protein ends would be oriented to the outside.

### Functional Characterization of AnCtr Mutants

**FIGURE 4:**
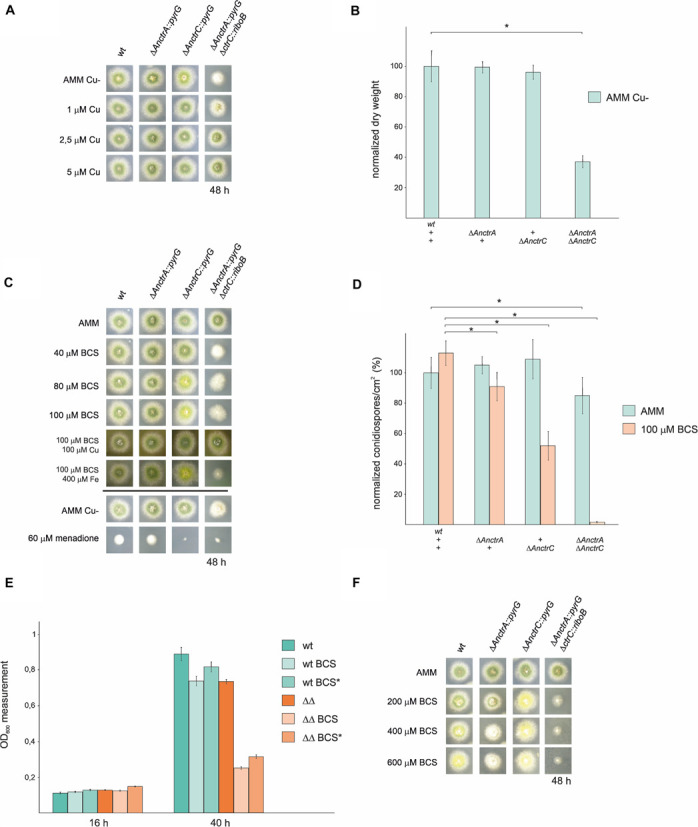
Functional analysis of *AnctrA* and *AnctrC* mutants. **(A)** Spores from the mutant strains were point-inoculated and images of colonies were taken after 48 h of incubation at 37°C in 0 μM Cu AMM (Aspergillus minimal medium) and 0 μM Cu AMM with 1, 2.5, and 5 μM Cu. **(B)** Dry weight measurements of the mutant strains incubated for 16 h at 37°C in 0 μM Cu AMM. **(C)** Spores from the mutant strains were point-inoculated and images of colonies were taken after 48 h of incubation at 37°C in AMM and AMM with 100 μM CuSO4, 100 μM BCS, 100 μM BCS with 100 μM CuSO4 and 100 μM BCS with 400 μM FeSO4. **(D)** Conidia production was determined by quantifying the number per unit of colony surface area (conidia/cm^2^). In order to facilitate data comparison, growth of the wt strain at basal level was designated 100%, data was normalized and presented as percentages.*Significant conidiospore number reduction *p* < 0.05. **(E)** In liquid medium culture experiments, 6 × 10^4^ spores of the WT and Δ*AnctrA*Δ*AnctrC* strains were inoculated for 16 h and 40 h at 37°C, under basal conditions (MMA), low copper availability conditions (BCS) and low copper availability conditions after 16 h of normal growth (BCS*). **(F)** Spores from the mutant strains were point-inoculated and images of colonies were taken after 48 h of incubation at 37°C in AMM and AMM with 200, 400, and 600 μM BCS.

The function of AnCtrA and AnCtrC through mutant analysis was recently reported (Cai et al., 2019); however, the individual role of each protein and their hierarchy in copper homeostasis remained unspecified. To gain further insight into this matter, single and double knock-outs of *AnctrA* and *AnctrC* were generated by gene replacement techniques (see list of strains). The capacity of these strains to grow at different copper availability conditions was tested. We first reproduced the experiment conducted by Cai et al. (2019), by testing all generated mutant strains in 0 μM Cu media with identical results: the single deletion mutants showed a WT-like phenotype and the only strain showing growth disruption was the double null mutant Δ*AnctrA*-Δ*AnctrC* (Figure 4A). These results provided evidence that under copper deficiency condition, AnCtrA and AnCtrC complemented each other. Addition of copper to the medium rescued the double deletion mutant defective phenotype. Similar results were obtained in liquid media as only the double null mutant showed a significantly lower growth rate than the WT strain (Figure 4B).

The above results showed that removing Cu from the trace element solution did not help discern phenotypic differences between single null mutants. Thus, we used the copper chelator bathocuproine disulfonic acid (BCS) in our experiments. As shown in Figure 4C, null mutant colonies did not exhibit appreciable alterations in radial growth or conidial pigmentation on regular solid minimal medium. Under copper starvation conditions (AMM supplemented with BCS), Δ*AnctrC* exhibited secondary level copper limitation effects at 80 μM concentration, displaying yellowish conidia which could be a result of the inactivity of the copper-dependent conidial laccase AnYA due to Cu deficiency. In contrast, the Δ*AnctrA* mutant presented no appreciable copper limitation effects. When both mutations were combined, primary level copper limitation effects were visible. Radial growth of the double knock-out strain was reduced to 40% and no pigmentation was appreciated. Upon Cu^+^ supplementation (100 μM BCS + 100 μM CuSO_4_), normal growth and morphology were restored. It has been described that copper and iron metabolism are related in fungi, and iron can recover Δ*mac*1 colony morphology -very similar to the double knock-out strain shown in Figure 4C- to some extent in *A. fumigatus* (Cai et al., 2017). Therefore, we tested whether addition of FeSO_4_ could rescue the colony defects observed in Δ*Anctr* mutants grown under copper starvation conditions. Fe supplementation (100 μM BCS + 400 μM FeSO_4_) did not rescue the defects in null mutants (Figure 4C). Since SODs are also copper dependent enzymes, we hypothesized that a menadione (a widely used oxidative stress inducing agent) experiment could probably help clarify the nature of the differences observed so far between the two transporter null mutants. However, with 60 μM menadione, the phenotype was similar; the Δ*AnctrC* strain was visibly more affected by the oxidative damage generated by menadione, suggesting that in the condition tested SOD copper supply depended more on AnCtrC.

In addition to the above colony growth inhibition and pigmentation defects, hyphal growth in liquid medium and conidia production were also studied. To investigate the effect of the deletion of the *Anctr* coding genes on conidial production, we harvested and compared the number of conidiospores produced by each strain, under basal and low copper availability conditions (Figure 4D). Under basal conditions, single deletion of *Anctr* genes had no apparent effect on conidia production. Under copper deficiency conditions *AnctrA* deletion had a slight effect, but *AnctrC* deletion had a remarkable impact; conidia numbers were halved by comparison to the wild-type strain. Numbers of conidia are connected with the amount of biomass and the Δ*AnctrC* colony grown in solid AMM medium with 100 μM BCS was not smaller than the colony grown in AMM. Thus, copper scarcity had no effect on colony expansion, but on colony density, an effect that has also been described under other stresses conditions such as carbon or nitrogen starvation (Richie et al., 2007). Deletion of both *Anctr* proteins had a slight effect at control conditions. Under copper starvation conditions, conidia quantity was, 37-fold lower than the wild-type strain (Figure 4D).

Hyphal growth studies were conducted with the WT stain and the Δ*AnctrA*-Δ*AnctrC* strain. At 16 h both strains presented very similar growth rates under all tested conditions. However, after 40 h, the double deletion strain presented a dramatically lower growth rate in low copper availability conditions compared to the WT and itself in basal copper conditions (28.1% and 35.2% compared to WT, for BCS and BCS^∗^ conditions, respectively) (Figure 4E). Thus, environmental copper deprivation had no apparent effect in the germination process but had a strong effect in the ensuing growth phase.

Since the addition of 100 μM BCS to media showed results for the *AnctrC* null mutant; however, but no characteristic phenotype with the null *AnctrA* mutant and the WT strain, we increased the BCS concentration (400–600 μM). In these extremely low copper availability conditions, the WT strain presented secondary copper limitation symptoms like pigmentation deficiency, similar to that of the Δ*AnctrC* strain. In addition, the Δ*AnctrA* strain displayed primary level copper limitation effects as a reduced radial growth and lack of pigmentation, similar to the double null strain albeit less aggravated (Figure 4F).

Taken together, the results support the interpretation that AnCtrA and AnCtrC are copper transporting proteins that complement each other. AnCtrC seems to be the principal copper transporting protein at nutritional and mild copper deficiency conditions, as the *AnctrC* deletion strain is more susceptible to oxidative damage and shows secondary copper limitation effects like defects in sporulation and spore pigmentation. However, the phenotypes of the WT strain and Δ*AnctrC* become very similar in extreme copper scarcity conditions, suggesting that the contribution of AnCtrC in those conditions is very limited. Although *AnctrA* deletion has no tangible effect under mild copper deficiency, primary level copper limitation effects are visible in extreme copper deficiency conditions. When both transporters were jointly deleted, pigmentation and colony growth defects were far greater than in the single null strains, as manifested by strongly reduced growth rate in liquid medium under low copper availability. This would support the view that both transporters cover an extended concentration range for environmental copper uptake.

### AnCtr Protein Expression Dynamics

After identifying the copper uptake proteins involved in copper homeostasis, their expression patterns were investigated, with special interest in AnCtrA, which remained uncharacterized. Hence, the expression profiles of AnCtrA and AnCtrC throughout Cu and BCS treatments, were examined in time course experiments.

**FIGURE 5:**
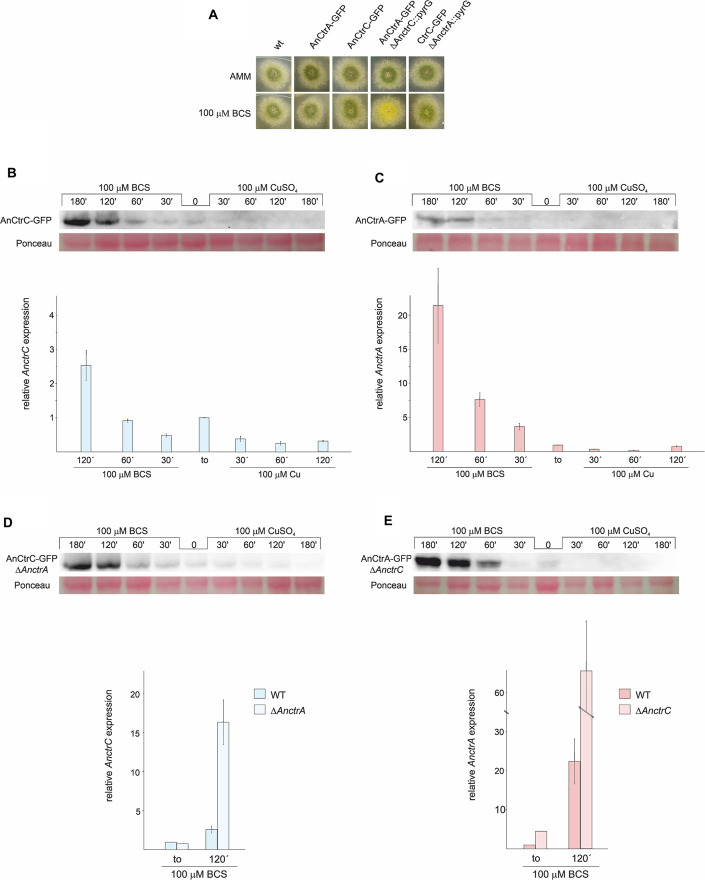
AnCtrC and AnCtrA expression profiles with Cu and BCS. **(A)** Phenotypes of the strains used for expression analysis of AnCtrC and AnCtrA in AMM and AMM + BCS media. **(B)** Western Blot and RT-PCR data showing changes in AnCtrC and *AnctrC* expression levels after addition of 100 μM CuSO_4_ or 100 μM BCS. **(C)** Western blot and RT-PCR data showing changes in AnCtrA and *AnctrA* expression levels after addition of 100 μM CuSO4 and 100 μM BCS. **(D)** Western blot and RT-PCR data showing changes in AnCtrC and *AnctrC* expression levels in a Δ*AnctrA* genetic background, after addition of 100 μM CuSO4 and 100 μM BCS. **(E)** Western Blot and RT-PCR data showing changes in AnCtrA and *AnctrA* expression levels in the Δ*AnctrC* genetic background, after addition of 100 μM CuSO4 and 100 μM BCS.

We generated two fluorescent protein-tagged chimera strains: AnCtrA:GFP and AnCtrC:GFP. In order to test whether GFP tagging could alter protein function we verified that both strains followed wild-type like phenotypes under BCS treatments in plate experiments (Figure 5A).

RT-PCR experiments revealed that Cu and BCS addition caused opposite effects on *AnctrC* expression (Figure 5B). Shortly after 100 μM CuSO_4_ addition, *AnctrC* transcript levels were reduced four-fold and this tendency was maintained throughout the experiment. On the other hand, upon addition of 100 μM BCS, initially presented lower relative expression of *AnctrC* transcript than at *t*_*o*_, followed by a gradual increase, reaching the highest level at 120′ (2.5-fold). Western blot analyses were coherent with RT-PCR data. When BCS was added to the medium, a 50 kDa band coinciding with the AnCtrC:GFP monomer was visible. The signal detected at short incubation times was similar to the one detected at *t*_*o*_. The signal grew stronger at longer exposures. After Cu addition, AnCtrC protein signal gradually faded through time.

The *AnctrA* transcript expression pattern showed a more specific pattern for copper deficiency conditions (Figure 5C). When copper was added, expression levels exhibited a five-fold decrease. However, upon BCS addition, expression increased up to 21.5-fold relative to the total RNA extract at *t*_*o*_, substantially greater than observed in *AnctrC.* Western Blot analyses exhibited a 47 kDa band only visible in samples treated with BCS. Although the observed band was very faint at early incubation times, it grew stronger with increasing incubation times.

Phenotypic observation of the mutants showed that AnCtrC is effective under copper sufficiency or and mild copper deficiency conditions, while AnCtrA function gains relevance under extreme copper deficiency conditions. Taking this into account, we decided to study the behavior of *AnctrA* and *AnctrC*, RNA and protein, in their respective null mutants and compared them with WT strain levels. *AnctrC* expression in a Δ*AnctrA* background, was slightly lower at basal conditions. However, at later times, expression level grew 16.74-fold higher under copper deficiency conditions (Figure 5D). At the protein level, the expression profile was maintained. *AnctrA* expression on the other hand, was clearly different in a Δ*AnctrC* background. At basal levels *AnctrA* expression was 4.5-fold higher and 63.81-fold higher under copper starvations conditions (Figure 5E). At the protein level, *t*_*o*_ protein signal could be detected and with BCS incubation, the signal became considerably stronger compared to that in the wild-type background.

The results from this study demonstrated that *AnctrA* and *AnctrC* expression is copper dependent, albeit with some differences (Figure 5). *AnctrC* expression profile is more stable through time in line with a possible role of copper supply in nutritional and mild copper deficiency conditions. *AnctrA* expression on the other hand, experiences an outstanding induction after BCS addition, a fitting expression pattern for an extreme copper deficiency copper transporter. The implications of these results will be further elaborated when the relative contribution of each protein is discussed.

### AnCtr Localization

**FIGURE 6:**
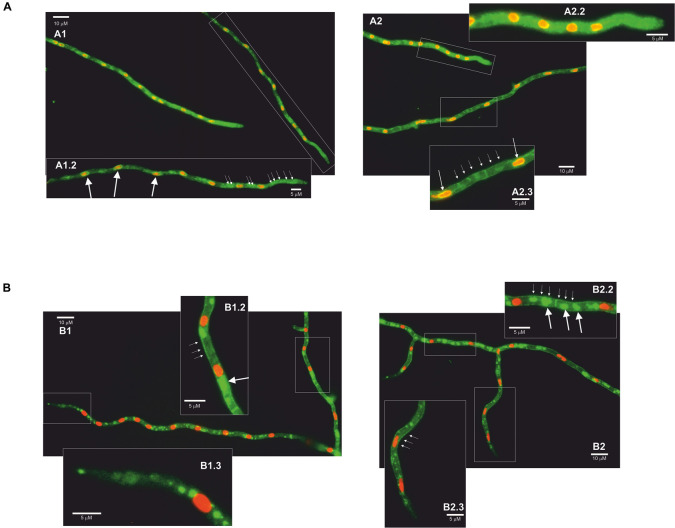
Fluorescence microscopy images of AnCtrC and AnCtrA chimeras. **(A)** AnCtrC:GFP-Hhoa:mRFP protein expressing strains were grown in selective WATCH medium for 16 h at 30°C. Images show the protein localization before BCS addition **(A1)** and 2 h after 100 μM BCS addition **(A2)**. Pictures A1.2–A2.2–A2.3 show a closer look at the most interesting features. **(B)** AnCtrA:GFP-HhoA:mRFP protein expressing strains were grown in selective WATCH medium for 16 h at 30°C. Images show the protein localization before BCS addition **(B1)** and 2 h after 100 μM BCS addition **(B2)**. Pictures B1.2–B1.3–B2.2–B2.3 show a closer look at the most interesting features.

AnCtrA and AnCtrC protein localization was conducted *in vivo* using strains expressing C-terminally GFP-tagged chimeric proteins, together with AnHhoA:mRFP marked strains. AnHhoA is Histone 1, a marker of the nuclear chromatin. Ctr proteins function as copper internalizing proteins. Considering the likely mobility upon changes in environmental copper concentration, we grew our GFP chimera expressing strains in regular WATCH medium, added BCS to the medium and observed protein dynamics within a 2 h period. In regular WATCH media without copper deprivation, AnCtrC:GFP was detected throughout the cytosol of hyphae (Figure 6A1). Many oval structures that colocalized with the red nuclei (white arrows) were distinguished, with some associated membranes displaying green fluorescence and the fluorescence signal was notably polarized (Figure 6A1.2). After BCS addition fluorescence signal became more visible in the plasma membrane but no protein migration was appreciated. In the vicinity of the tip area, no fluorescence was localized in the plasma membrane and the signal inside the hyphae polarized toward the tip. After 2 h of BCS addition the presence of the protein in the plasma membrane became clearer along the hyphae but not in the tip region (Figure 6A2). Fluorescence signal was notably polarized in the tip region (Figure 6A2.2). Fluorescence was still visible in the perinuclear area and discrete fluorescence signals were detected elsewhere within hyphae but the identity of the organelles could not be specified (Figure 6A2.3). In the case of AnCtrA, throughout the course of the experiment, a gradual increase in fluorescence intensity was observed but protein localization remained quite stable. Without copper deprivation, the protein was mainly localized in irregular granules dispersed along the hyphae (Figure 6B1). Disperse fluorescence patches could be detected in the plasma membrane (Figure 6B1.2) and as with AnCtrC, close to the tip region no protein was localized in the membrane (Figure 6B1.3). After 2 h of BCS addition, the signal in the plasma membrane became more notorious (Figure 6B2). Besides the plasma membrane fluorescence signal numerous other structures were visible along the hyphae, possibly early endosomes (Figure 6B2.2). Even in copper deprivation conditions, no protein was localized in the tip region (Figure 6B2.3). The subcellular localization of each protein will be further discussed.

In conclusion, in case of AnCtrC, the signal accumulated around nuclei associated membranes, most likely the nuclear envelope (Markina-Inarrairaegui et al., 2013) and other structures; considering the polarization of the fluorescence signal possibly the peripheral ER. In low copper availability conditions, the fluorescence signal along the hyphae, except for the tip, partially shifted toward the membrane; nevertheless, most of the protein was still detected along the hyphae. In the case of AnCtrA the fluorescence signal was in the plasma membrane, but toward the tip region of the hyphae, the plasma membrane appeared free of fluorescence. The protein could be also detected in numerous morphologically heterogeneous compartments. These could be vacuolar compartments or early endosomes, considering the similarities between the role and expression pattern of AnCtrA and previously described copper uptake proteins.

### C-Terminal Mutations of AnCtrA and AnCtrC

**FIGURE 7:**
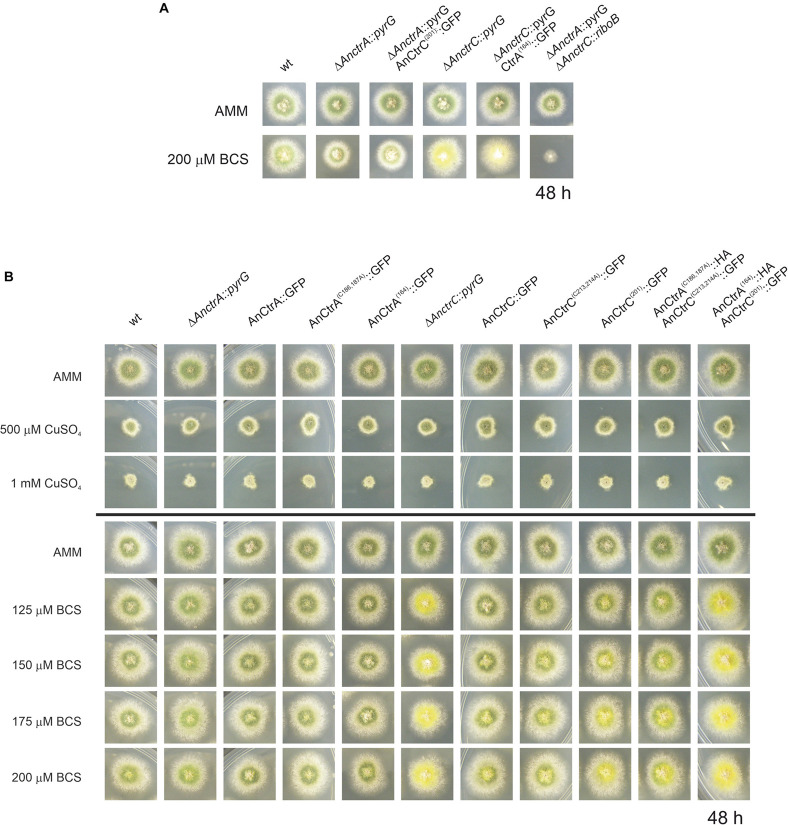
Functional analysis of AnCtrA and AnCtrC C-terminal mutants. **(A)** Phenotypes of the AnCtrC and AnCtrA C-terminal mutants in AMM and AMM + BCS media to assess protein function. **(B)** Spores from the mutant strains were point-inoculated and images of colonies were taken after 48 h of incubation at 37°C in AMM (Aspergillus minimal medium) and AMM with different concentrations of CuSO_4_ and BCS.

In organisms like *S. cerevisiae* and humans, Ctr protein C-terminals reportedly play a crucial role in shutting down copper uptake when intracellular copper concentrations reach a threshold (Schuller et al., 2013). Certain copper binding residues, namely cysteines and histidines, are specifically involved in the mechanism, which is separate from copper uptake (Wu et al., 2009; Clifford et al., 2016). In the C-terminal of AnCtrA and AnCtrC there are two adjacent cysteines that are conserved in many other orthologs, flagging them as copper binding residues. In order to test this hypothesis, single and double mutants of the mentioned cysteine residues AnCtrA^*C*186,187*A*^ and AnCtrC^*C*213,214*A*^ were generated by alanine substitution. C-terminally truncated mutants, AnCtrA^164^ and AnCtrC^201^, were also generated, in order to check if there was any difference in copper susceptibility compared to the cysteine mutants. The mutants were checked for a possible loss of function due to the inserted mutations. All mutants were proven to be functional, as the outcome of the C-terminal mutation in combination with a Ctr deletion was not similar to the double null mutant (Figure 7A). The mutants were grown in AMM with different concentrations of CuSO_4_ and BCS as shown in Figure 7B.

Neither the deletion of the C-terminal domain nor the mutation of the cysteine residues resulted in added susceptibility toward copper. However, the mutants presented secondary level copper limitation effects in copper scarcity conditions, especially the double C-terminal deletant strain. The AnCtrA^(164)^:HA-AnCtrC^(201)^:GFP strain phenotype was similar to the Δ*AnctrC* phenotype regarding conidial pigmentation deficiency at 125 μM BCS. The AnCtrC^(201)^:GFP and AnCtrA^(C186,187*A)*^:HA-AnCtrC^(C213,214*A)*^:GFP also showed pigmentation deficiencies but to a lower degree. The C-terminally truncated mutants were still functional as shown in Figure 7A.

Therefore, the broadly conserved di-cysteine motif was shown to have no impact on copper uptake shut-down under the conditions tested. However, this motif may be involved in copper uptake, since removing the C-terminal and the cysteine residues does appear to reduce uptake capacity as their deletion results in defective pigmentation, a secondary level copper limitation effect. This would mean that the copper uptake regulation mechanism of the *A. nidulans* Ctr proteins is different from the so far studied regulation mechanisms and that the contribution of the C-terminal is necessary for full capacity copper uptake.

### Screening for Cupric Metalloreductases

**FIGURE 8:**
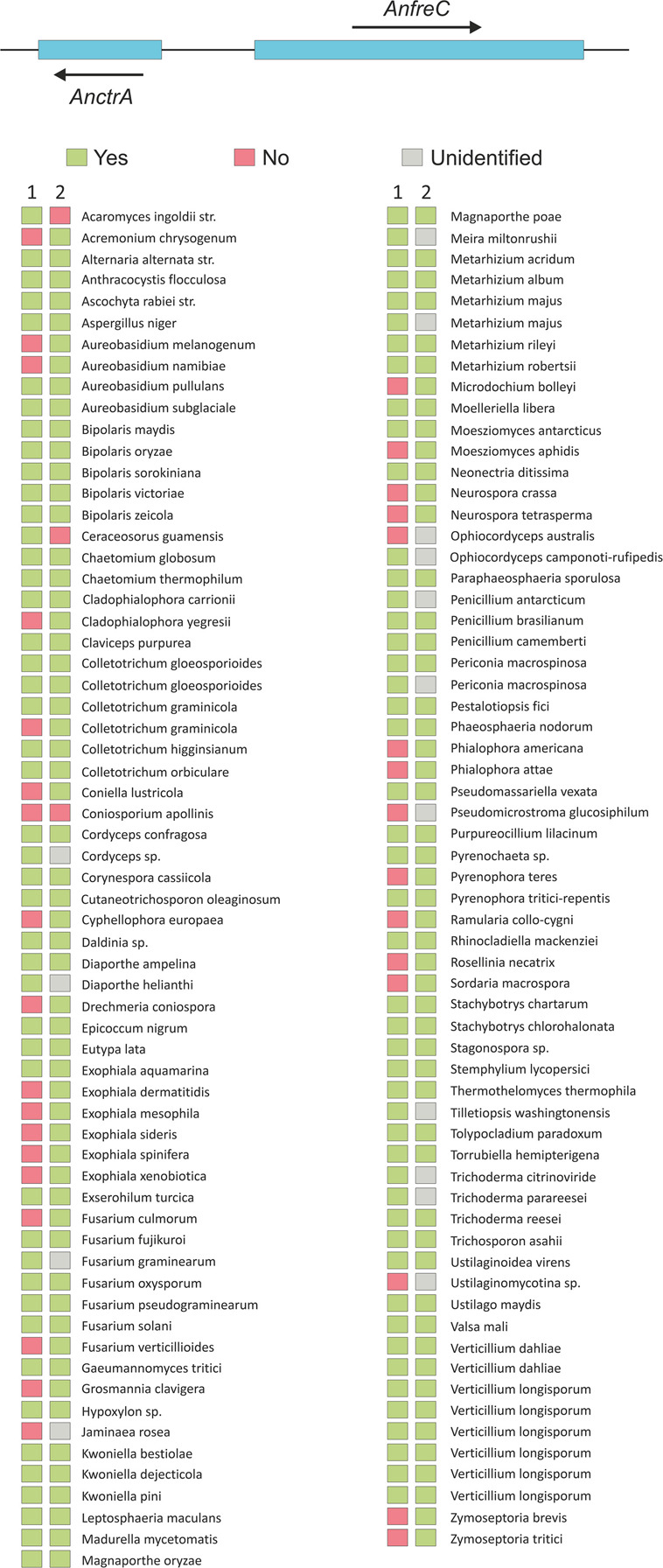
An image representing the chromosomal location of *AnfreC*-*AnctrA*, and a list of fungal organisms that possess an *AnctrA* ortholog are presented. (1) The organism possesses another Ctr protein ortholog of *AnctrC*. (2) The organism possesses an Ox-Red coding gene beside the *AnctrA* ortholog, just like *A. nidulans*. The color of the rectangles next to the organism represents: green (yes), red (no) and gray (unidentified).

As mentioned in the RNA-seq analysis, RNA-sec offered the opportunity to search for Plasma Membrane metalloreductases, homologs to the Fre protein family. The expression of the copper metalloreductases reportedly follow the same expression pattern as Ctr proteins since both are under control of the TF Mac1 (Yamaguchi-Iwai et al., 1997; Levitin and Whiteway, 2007). Our screen retrieved three candidates that were co-regulated with the Ctr protein out of ten members of the family of metalloreductases present in the *A. nidulans* genome: *AnfreA*, AN0773, and AN3208. *AnfreA* is a characterized iron reductase and it has no putative AnMac1 binding sequence. AN0773 and AN3208 are two uncharacterized putative copper reductases that possess a possible AnMac1 binding region in their respective promoter regions. However, a detail caught our attention. AN3208 is a putative cell surface metalloreductase adjacent to AnctrA and they share the promoter region where AnMac1 binds (Figure 4). This feature reinforces the idea that it may be a copper metalloreductase. Thus, it was named AnfreC. Synteny and multiple alignment data in the EnsemblFungi site revealed that the *AnctrA-AnfreC* ortholog’s position and orientation is conserved in the majority of fungal species that contain an *AnctrA* ortholog. The majority of screened species (110/127) contain the putative plasma membrane high affinity copper transporter and a metalloreductase located in tandem (Figure 8).

In summary, AN0773 and *AnfreC* fulfill the conditions to be copper metalloreductases. However, due to the unique features of *AnfreC* mentioned above it is a suitable candidate to be the main cupric metalloreductase in *A. nidulans*.

## Discussion

This study provides additional information on the expression and function of the high affinity copper uptake system reported in yeast and filamentous fungi of clinical interest. The study of gene expression profiles under basal copper availability conditions, copper deprivation and copper toxicity has shed new light on the overall adaptation of *A. nidulans* to copper fluctuations, as well targeting the dynamics of copper transporters. The subsequent analysis through targeted mutagenesis of the principal copper transporters have enabled an interpretation of their individual contributions in the copper uptake process, their expression patterns and their subcellular localization dynamics. In addition, new inquiries into the role of transporter C-terminal domains together with the identification of putative membrane copper reductases have been initiated.

The response to copper toxicity in *A. nidulans* involves the differential expression of 13.7% of its genes. Significant changes were recorded on recognized copper homeostasis genes. Even though the expression of the TFs regulating the process was not significantly altered, that of proteins responsible of copper uptake and detoxification was very notable. The gene coding for the copper exporting P-type ATPase AnCrpA (Antsotegi-Uskola et al., 2017), was up-regulated. On the other hand, the two characterized copper transporter genes *AnctrA* and *AnctrC* (Cai et al., 2019) were down-regulated, in line with the objective of reducing the copper uptake to the minimum. Finally, the notable upregulation of the intracellular copper trafficking P-type ATPase *AnygA* (Clutterbuck, 1990) might be a response to the presence of high amounts of copper ions within the cell that have to be distributed or compartmentalized. Out of the identified three putative copper chaperones only the putative Cu/Zn SOD chaperone AN6045 was significantly up-regulated. The functional counterpart in *S. cerevisiae*, chaperone Atx1, responsible for copper distribution within the cell, binds excess cytosolic copper (Lin and Culotta, 1995) and could act as a first level scavenger at low levels of toxicity. This effect may have been relevant under our experimental conditions, as discussed below.

Free cytoplasmic copper is known to produce ROS through Fenton-type reactions (Sutton and Winterbourn, 1989; Timoshnikov et al., 2019). Superoxide dismutases, accept electrons from reduced metals or superoxides, yielding hydrogen peroxide, which, along with small organic peroxides, is the substrate of catalases (2 H_2_O_2_ → 2 H_2_O + O_2_) (Zamocky et al., 2008). Hence, the reported copper toxicity response involves the activation of both groups of enzymes (Wiemann et al., 2017). In our experiments, however, we observed that catalases *AncatB* (2.82-fold), *AncatC* (2.36-fold) and *AncpeA*/*AncatD* (2.59-fold) were up-regulated, but the expression of the Cu/Zn superoxide dismutase *AnsodA* (Holdom et al., 1996) (reported in Table 1), an enzyme activated in response to copper and zinc-derived ROS toxicity, remained unchanged, along with that of the ROS responsive transcription factors *AnatfA* and *AnnapA* (Table 2). These variations may stem from the differences in copper concentrations and the relative sensitivities of the strains used by Wiemann et al. (2017) and this study. In our experiments, 100 μM Cu concentrations were used, a dose that showed no visible effect in *A. nidulans* colony development (Antsotegi-Uskola et al., 2017). On the other hand, Wiemann et al. (2017) used a 200 μM copper concentration, in an organism at which 50 μM already compromised colony development. It is therefore likely that, under conditions which elicit no phenotypic symptoms of copper toxicity, the AnCrpA detoxification system could have removed the free cytosolic copper, rendering ROS neutralization unnecessary. On the other hand, Wiemann et al. (2017) combined higher copper concentrations and greater susceptibility may have overwhelmed the capacity of AfCrpA, thus triggering the upregulation catalases to quench ROS and H_2_O_2_. Both results may thus depict the response to two different levels of copper toxicity. We hypothesize that catalases are heme-containing enzymes (Scherer et al., 2002) and it is well known that intracellular free copper ions can displace protein-bound iron. The selective up-regulation of the catalases in this case may be a response to this phenomenon, leading to the greater demand for functional catalases. More evidence will be required to confirm this interpretation.

Aside from catalases, the copper toxicity response also modified the expression of many other oxidoreductases. Laccases are blue–copper enzymes involved in lignin degradation or pigment biosynthesis (Scherer and Fischer, 2001). However, laccases don’t show a specific expression pattern in response to copper toxicity (Table 1). Other oxidoreductases corresponded to membrane function-related metabolic processes such as sterol metabolism. Sterols, especially ergosterol, are the most abundant lipids in fungal cell membranes (Rodrigues, 2018), with roles in integral protein stabilization, membrane fluidity and permeability (Alvarez et al., 2007; Abe and Hiraki, 2009). Under copper toxicity, the expression of proteins involved in transmembrane traffic augmented; for example, many putative sugar transporters were up-regulated. This could be ascribed to a heightened requirement for energy (ATP) and reducing power (NADH), both principal products of aerobic sugar catabolism. For example, the ATP-dependent copper detoxification protein AnCrpA was highly up-regulated in this study, in line with earlier observations (Antsotegi-Uskola et al., 2017). Changes in differential expression of various heavy metal transporters may indicate that a change of copper concentration could cause an imbalance in heavy metal homeostasis, as described in *A. fumigatus* (Cai et al., 2017). Finally, a stress mechanism with such a wide ranging impact on cellular metabolism should also alter the regulation of a core regulatory process as transcription. A TF that experienced notable lower expression under copper toxicity conditions was *AnhapX* and this, in turn, is consistent with the down-regulation of the siderophore biosynthesis cluster that it has been reported to regulate (Schrettl et al., 2010). The same result was obtained with the antimicrobial compound emericellamide biosynthesis cluster (Table 2). Finally, analyzing the data of the RNA-seq we found out a highly up-regulated set of genes. The upregulation of an *N*-acetylglucosamide transporter, AN2562, could be an adaptive response to the high copper environment as this compound proves to be important for survival in the phagosome (Vesely et al., 2017) where copper toxicity is used to kill by the innate immune system (Li et al., 2019). Secondly, it is very convenient that a cysteine and methionine transporter, AN2560, would be upregulated in such conditions as they are two amino-acids that participate in copper binding motifs (Pena et al., 2000). The Flavin-containing monooxygenase might play an important role in creating an optimal redox environment threatened by the presence of copper in the cytosol (Suh et al., 2000). These three genes together with the predicted oxidoreductase AN2559 and the putative methyltransferase AN2561 might comprise a defensive gene cluster against copper toxicity.

The phylogenetic characteristics of two *A. nidulans* Ctr proteins, AnCtrA and AnCtrC, have been examined in this study. Although the reference Ctr sequence used to screen for orthologs in fungi had been ScCtr1, none were found in the *A. nidulans* genome with that criterion. According to the Ensembl fungi ortholog predictions, homologs of ScCtr1 are only found in the Saccharomycetales order. Moreover, most Ctr proteins of filamentous fungi shared higher similarity with ScCtr3 (Figure 2). Unfortunately, *Scctr3* is poorly documented as it is disrupted by a Ty2 transposon in many laboratory strains (Knight et al., 1996). AnCtrC showed phylogenetic relationship with numerous Ctr orthologs in *A. fumigatus*, *S. cerevisiae* and various other fungi. On the other hand, AnCtrA showed similarity with other putative Ctr proteins that have not been characterized.

Both proteins possess the characteristic features of Ctr proteins (Zhou et al., 2003; Petris, 2004; Rees and Thiele, 2004). Ctr family members generally harbor three putative TMDs (Puig et al., 2002), but according to topology software predictions, AnCtrA has only two TMDs. This has also been reported in *A. fumigatus* and *C. neoformans* (Waterman et al., 2012; Park et al., 2014). Cysteine residues are present throughout the sequence and cysteines are known for their affinity to copper (Pena et al., 2000). Both proteins contain two cysteine residues flanking the methionine residue adjacent to TMD1. This soft Lewis base may be important to coordinate copper since it has been described that metal binding residues located close to the plasma membrane seem to play a more important role in copper transport (Puig et al., 2002).

Functional characterization analyses in this study showed that the two Ctr proteins worked independently and the range of copper concentrations they covered were partially complementary, as derived from the obtained phenotypes. The phenotypes observed were broadly interpreted in terms of acute or partial deficiencies as follows. Normal growth rates and WT-like green conidial pigmentation were interpreted as an indication of adequate copper supply for basic growth and developmental functions. Defects in spore pigmentation and sporulation that did not affect growth rate were interpreted as the result of a partial limitation in copper. Conidiogenesis is strongly reliant on autophagy-based resources (Kikuma et al., 2007; Richie et al., 2007). The yellowish spore pigmentation could be explained by the inactivity of the copper-dependent conidial laccase AnYA; the phenotype corresponds with the *yA2* phenotype (Hermann et al., 1983; Scherer and Fischer, 2001). On the other hand, acute copper deficiency was interpreted to affect extracellular copper transport with direct consequences on vegetative growth. Following these criteria, we surmised that AnCtrC functions as the principal copper uptake protein under copper sufficiency and mild copper deficiency conditions. AnCtrA may complement the lack of AnCtrC at moderate copper deficiencies but is specifically required for extreme copper deficiency scenarios.

As shown in this study, conditions of low copper availability induce a gradual up-regulation in Ctr expression, while high copper levels induce the opposite effect. The rapid shutdown of the copper uptake system under high extracellular copper concentrations is of great importance to avoid copper toxicity (Bertinato and L’Abbe, 2004; Dong et al., 2013). The expression patterns of AnCtrA and AnCtrC under similar testing conditions share certain similarities with *S. cerevisiae* Ctr proteins; a Ctr protein with a more stable expression pattern (ScCtr3) and another one that is uniquely expressed under low copper availability conditions (ScCtr1) (Pena et al., 2000). AnCtrC is the only high-affinity copper transporter expressed at basal copper levels, which means it is likely to cover for copper intake under ordinary copper availability (Figure 5B). On the other hand, AnCtrA expression pattern seems to be designed for extreme copper scarcity conditions, when AnCtrC transport cannot meet the cell’s copper requirements. These results are in line with the conclusions from phenotypic analyses of selective null mutants discussed above.

In terms of protein localization dynamics, Ctr proteins are known to follow a very dynamic trafficking pathway. Pena et al. (2000) described that ScCtr3 traveled to the plasma membrane through the Trans-Golgi network and the fluorescence signal of tagged proteins accumulated in the ER after inhibiting protein traffic from ER to Golgi. However, when copper concentration rose, Ctr proteins like ScCtr1, moved from the plasma membrane to the vacuole (Liu et al., 2007), or early endosomes, as in the case of hCtr1 (Eisses and Kaplan, 2002; Clifford et al., 2016). The predominant ER localization of AnCtrC (Markina-Inarrairaegui et al., 2013) and its partial migration to the plasma membrane in copper deficiency conditions support its role as a copper uptake protein covering transport under copper sufficiency or mildly limiting conditions. The predominant plasma membrane and vesicular localization of AnCtrA is in line with reported data of other proteins that respond to extreme copper deficiency scenarios such as described for ScCtr1 or hCtr1 (Eisses and Kaplan, 2002; Liu et al., 2007; Clifford et al., 2016).

The shutdown of copper transport as a measure to avoid toxicity has been documented many instances. Previous reports on this toxicity-preventing mechanism refer to certain residues, mostly cysteines, in the C-terminus of Ctr proteins as key elements in uptake modulation (Liu et al., 2007; Wu et al., 2009; Schuller et al., 2013). Copper has been postulated to bind C-terminal cysteines, purportedly triggering the mechanism. The possible role of the Ctr proteins’ C-terminal in activity modulation has not been studied in filamentous fungi. However, the C-terminal di-cysteine motif, a common heavy metal binding sequence (Lekeux et al., 2018) can be found in Ctr proteins of *Alternaria alternata*, *Aspergillus fumigatus*, *Botrytis cinerea*, *Fusarium graminearum*, *Histoplasma capsulatum* and *Magnaporthe oryzae*. We first hypothesized that this di-cysteine motif could act as copper uptake modulator, as the ScCtr1 C-terminal cysteine residues or the hCtr1 HCH motif. However, our results do not indicate any copper dependent uptake modulation or internalization role. Moreover, unlike the C-terminus of ScCtr1, that is dispensable for full copper uptake capacity (Wu et al., 2009), deletion of AnCtrC C-terminus results in secondary copper deficiency effects manifested as a pigmentation deficiency (Figure 2B). Thus, the C-terminus of the Ctr proteins plays some role in the copper uptake process. Further studies will be needed to clarify this question.

Plasma membrane metalloreductases are crucial components of the copper uptake process as they provide Ctr transporting proteins with the substrate in a transport-compatible reduced state (Labbe et al., 1997; Petris, 2004). There are documented reductases that reduce iron and also copper (Georgatsou et al., 1997); however, *AnfreA* was discarded for lacking a putative Mac1 binding sequence. *AN0773* and *AnfreC* both shared copper reductase features, but the phylogenetically preserved genomic location of *AnfreC* in tandem with *AnctrA* tipped the scales in its favor. The genes coding for the reductive iron assimilation complex composed by the oxidoreductase FetC and the iron transporter FtrA (Schrettl et al., 2004) are located in tandem, exactly like *AnfreC*-*AnctrA*, in Fusarium species (Park et al., 2007; Wiemann et al., 2012) and other Aspergillus species like *A. fumigatus, A. clavatus* and *A. fischeri* for example. This fact points out to *AnfreC* as the main copper oxidoreductase.

In conclusion, the RNA-seq experiments performed in this study have clearly shown that copper concentration has a notable impact on many cellular processes, especially the redox-state and transmembrane transport. The absence of phenotypic impact of the treatments used on colony development suggests that the concentration used for the RNA-seq experiment is within the range of concentrations covered by the P-type ATPase AnCrpA. The deep functional characterization of AnCtrA and AnCtrC has revealed that both Ctr proteins function in a complementary manner but each protein has a role in copper uptake. AnCtrC works in copper uptake under copper sufficiency conditions and AnCtrA is only activated under extreme copper deficiency. Both proteins display a copper-dependent expression but with different patterns that reinforce the above-mentioned hypothesis. Interestingly, both proteins are upregulated when the counterpart is missing but they are not able to fully complement each other. Both proteins are partially located in the plasma membrane; nevertheless, they are clearly dynamic proteins. The C-terminus CC motif neither the C-terminus itself are part of the copper uptake modulation mechanism in copper toxicity conditions but they do have a role in copper uptake. Finally, the putative plasma membrane metalloreductase *AnfreC* possesses very interesting features that make it a good candidate to be the main cupric metalloreductase.

## Data Availability Statement

The datasets presented in this study can be found in online repositories. The names of the repository/repositories and accession number(s) can be found below: https://www.ncbi.nlm.nih.gov/, PRJNA623550.

## Author Contributions

MA-U designed and conducted the experimental work, analyzed and interpreted the results, wrote the manuscript, and ensured the accuracy of the project as whole. AM-I made the concept and designed the work, analyzed data and contributed to the writing of the manuscript. UU con-conceived the work, ensured the scientific issue was appropriately investigated, ensured the integrity of the work, revised and approved the final version to be published. All authors contributed to the article and approved the submitted version.

## Conflict of Interest

The authors declare that the research was conducted in the absence of any commercial or financial relationships that could be construed as a potential conflict of interest.

## Footnotes

1 http://www.ebi.ac.uk/Tools/msa/clustalomega/

2 http://mobyle.pasteur.fr/

3 https://www.megasoftware.net/home

4 http://www.aspergillusgenome.org/download/sequence/A_nidulans_FGSC_A4/archive/

5 http://www.aspgd.org/download/go/gene_association.aspgd.gz

6 http://www.geneontology.org/ontology/obo_format_1_2/gene_ontology_ext.obo

7 http://www.bioconductor.org/packages/release/bioc/html/topGO.html

8 http://www.python.org/

9 http://rpy.sourceforge.net/rpy2.html

10 http://imagej.nih.gov/ij

## Supplementary Material

Supplementary Figure 1 Southern-blot validation of the mutant strains generated in this study. AnHhoA:mRFP strains were validated by microscope.
