# Supplementary figures and images for: Copper Homeostasis in Aspergillus nidulans Involves Coordinated Transporter Function, Expression and Cellular Dynamics
Source: Front Microbiol. 2020 Nov 17;11:555306. doi: 10.3389/fmicb.2020.555306 (PMC7705104; doi:10.3389/fmicb.2020.555306)

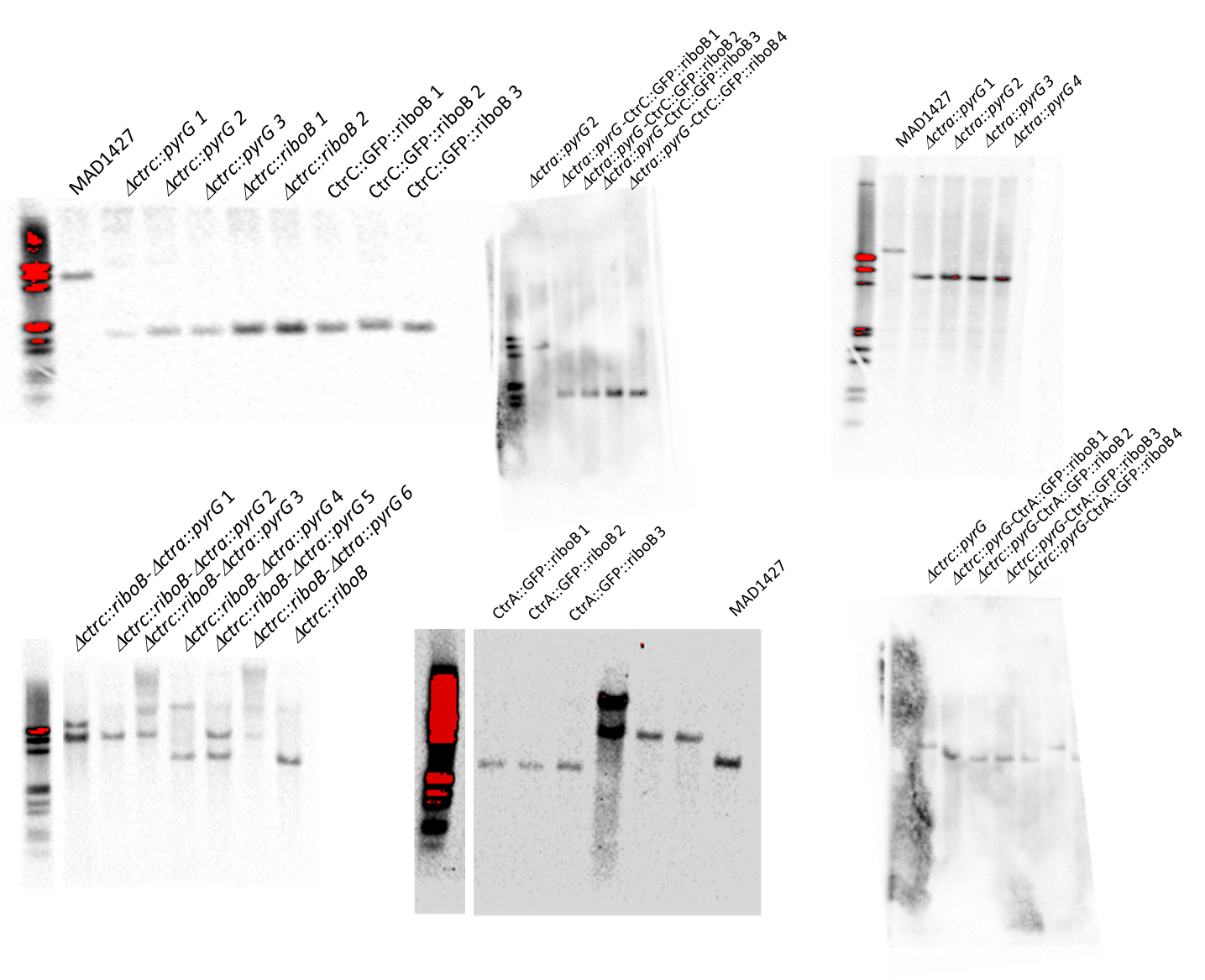

Supplement: Supplementary Figure 1 — Southern-blot validation of the mutant strains generated in this study. AnHhoA:mRFP strains were validated by microscope. [file Image_1.TIF]
