# Supplementary material for: Copper Homeostasis in Aspergillus nidulans Involves Coordinated Transporter Function, Expression and Cellular Dynamics
Source: Front Microbiol. 2020 Nov 17;11:555306. doi: 10.3389/fmicb.2020.555306 (PMC7705104; doi:10.3389/fmicb.2020.555306)
Supplement: Supplementary file 2 [file Table_1.DOCX]

**Table S1.** A*spergillus nidulans* strains used in this study.

| **Strains** | **Genotype** | **Reference** |
| --- | --- | --- |
| MAD1427 | *pyrG89, pabaA1; argB2; ΔnkuA::argB ; veA1, riboB2* | TN02A25 Oakley B. |
| MAD2731 | *pabaA1; argB2; ΔnkuA::argB ; veA1, riboB2* | Markina-Iñarrairaegui *et al.* (2011) |
| MAD2733 | *pabaA1; argB2; ΔnkuA::argB ; veA1* | Markina-Iñarrairaegui *et al.* (2011) |
| BD874 | *pyrG89, pabaA1; ΔctrC::pyrG^Af^; argB2; ΔnkuA::arg; veA1, riboB2* | This study |
| BD872 | *pyrG89, pabaA1; ΔctrC::riboB^Af^; argB2; ΔnkuA::arg; veA1, riboB2* | This study |
| BD878 | *pyrG89, pabaA1; ctrC::gfp::riboB^Af^; argB2; ΔnkuA::arg; veA1, riboB2* | This study |
| BD1160 | *pyrG89, pabaA1; ctrC::gfp::riboB^Af^; argB2; ΔnkuA::arg; ΔctrA::pyrG^Af^; veA1, riboB2* | This study |
| BD880 | *pyrG89, pabaA1; argB2;ΔnkuA::argB;ΔctrA::pyrG^Af^; veA1, riboB2* | This study |
| BD1158 | *pyrG89, pabaA1; argB2; ΔnkuA::argB; ctrA::gfp::riboB^Af^; veA1, riboB2* | This study |
| BD1162 | *pyrG89, pabaA1; ΔctrC::pyrG^Af^; argB2; ΔnkuA::arg; ctrA::gfp::riboB^Af^; veA1, riboB2* | This study |
| BD1064 | *pyrG89, pabaA1; ΔctrC::riboB^Af^; argB2; ΔnkuA::arg;ΔctrA::pyrG^Af^; veA1, riboB2* | This study |
| BD1361 | *pyrG89, pabaA1; ctrC*^C213A,C214A^*::gfp::riboB^Af^; argB2; ΔnkuA::arg; veA1, riboB2* | This study |
| BD1363 | *pyrG89, pabaA1; ctrC^201^::gfp::riboB^Af1^; argB2; ΔnkuA::arg; ctrA::gfp::riboB^Af^; veA1, riboB2* | This study |
| BD1357 | *pyrG89, pabaA1; argB2; ΔnkuA::arg; ctrA*^C186A,C187A^*::gfp::riboB^Af^; veA1, riboB2* | This study |
| BD1359 | *pyrG89, pabaA1; argB2; ΔnkuA::arg; ctrA^164^::gfp::riboB^Af^; veA1, riboB2* | This study |
| BD1365 | *pyrG89, pabaA1; ctrC*^C213A,C214A^*::gfp::riboB^Af1^; argB2; ΔnkuA::arg; ctrA*^C186A,C187A^*::ha::pyrG^Af^; veA1, riboB2* | This study |
| BD1367 | *pyrG89, pabaA1; ctrC^201^::gfp::riboB^Af1^; argB2; ΔnkuA::arg; ctrA^164^::ha::pyrG^Af^; veA1, riboB2* | This study |
| BD1369 | *pyrG89, pabaA1; ctrC::gfp::riboB^Af1^; argB2; ΔnkuA::arg; h1::mRFP::pyrG^Af^; veA1, riboB2* | This study |
| BD1371 | *pyrG89, pabaA1; argB2; ΔnkuA::arg, ctrA::gfp::riboB^Af^, h1::mRFP::pyrG^Af^; veA1, riboB2* | This study |
