# Supplementary material for: Copper Homeostasis in Aspergillus nidulans Involves Coordinated Transporter Function, Expression and Cellular Dynamics
Source: Front Microbiol. 2020 Nov 17;11:555306. doi: 10.3389/fmicb.2020.555306 (PMC7705104; doi:10.3389/fmicb.2020.555306)
Supplement: Supplementary file 3 [file Table_2.docx]

**Table S2.** Oligonucleotides used in this study.

| **Oligo** | **Sequence (5´-3´)** | **Utility** |
| --- | --- | --- |
| CtrA-gsp1 | GTTTGGCGTGTTGAAGTG | *ctrA* 5´ UTR |
| CtrA -gsp2 | CTTGTCGAATGTCTGGATAC | *ctrA* 5´ UTR |
| CtrA -gsp3 | CTTTGCTGTATATATTTTAAATGAAGCGC | *ctrA* 3´ UTR |
| CtrA -gsp4 | GCTTATTTCACAGATTGGGATTTCC | *ctrA* 3´ UTR |
| CtrA -gsp5 | AGTCTTCAAGAAGCGCTTTATCATTCT | *ctrA* 3´ ORF end |
| CtrA -gsp6 | GCCACAACACTTGGTCGCTTCCTC | *ctrA* 3´ ORF end |
| CtrA -gsp2* | GTATCCAGACATTCGACAAGACCGGTCGCCTCAAACAATGCTCTTCACCCTC | pyrG*^Af^* cassette |
| CtrA -gsp3* | GCGCTTCATTTAAAATATATACAGCAAAGGTCTGAGAGGAGGCACTGATGC | pyrG*^Af^* /gfp::riboB*^Af^* cassette |
| CtrA -gsp6* | GAGGAAGCGACCAAGTGTTGTGGCGGAGCTGGTGCAGGCGCTGGAGCC | gfp:: riboB*^Af^* cassette |
| CtrC-gsp1 | GGGAGTGATAGGTAAAAACA | *ctrC* 5´ UTR |
| CtrC -gsp2 | CTTGTTAATGTCGTTTGTTG | *ctrC* 5´ UTR |
| CtrC -gsp3 | TGTTTCACATACGCATATAA | *ctrC* 3´ UTR |
| CtrC -gsp4 | ATCCTCTTTCATCTCTTTCC | *ctrC* 3´ UTR |
| CtrC -gsp5 | CGTGCATCCAGTTCCAGCCTATCCATTC | *ctrC* 3´ ORF end |
| CtrC-gsp6 | ACCGCAGCATTTCGTAACAGCCGTTGC | *ctrC* 3´ ORF end |
| CtrC -gsp2* | CAACAAACGACATTAACAAGACCGGTCGCCTCAAACAATGCTCTTCACCCTC | pyrG*^Af^* /riboB*^Af^* cassette |
| CtrC -gsp3* | GGATCTGAATTATATGCGTATGTGAAACACTGTCTGAGAGGAGGCACTGATG | pyrG*^Af^* /riboB*^Af^* /gfp:: riboB*^Af^* cassette |
| CtrC -gsp6* | GCAACGGCTGTTACGAAATGCTGCGGTGGAGCTGGTGCAGGCGCTGGAGCC | gfp:: riboB*^Af^* cassette |
| CtrA-fw | CTCTGTCGAGATGCTCTGGAACTG | *ctrA* forward primer for RT-PCR |
| CtrA- rev | GCAGGCTCTCTTCGTAATCCTTGG | *ctrA* reverse primer for RT-PCR |
| CtrC-fw | GTGTCATCTCGATGCTGTGGAA | *ctrC* forward primer for RT-PCR |
| CtrC- rev | CGAGGACTATGACGAGGCAGATG | *ctrC* reverse primer for RT-PCR |
| BenA-fw | AGATGCGCAACATCCAGAGC | *benA* forward primer. RT-PCR control |
| BenA-rev | CTGGTACTCGGAGACGAGATCG | *benA* reverse primer. RT-PCR control |
| CtrC-AA gsp 3 | GCTGTTACGAAAGCCGCCGGTGGAGCTGGT | *ctrC* fordward primer for Cysteine mutation |
| CtrC-AA gsp 3 | ACCAGCTCCACCGGCGGCTTTCGTAACAGC | *ctrC* reverse primer for Cysteine mutation |
| CtrC C-term gsp 3 | CTCGTGGGAGTACATTGGGGGTGGAGGAGCTGGTGCAGGCGCTGGAGCCG | *ctrC* forward primer for C-term mutation |
| CtrC C-term gsp 6 | TCCACCCCCAATGTACTCCCACGAG | *ctrC* reverse primer for C-term mutation |
| CtrA-AA gsp 3 | GAAGCGACCAAGGCTGCTGGCGGAGCTGGT | *ctrA* fordward primer for Cysteine mutation |
| CtrA-AA gsp 3 | ACCAGCTCCGCCAGCAGCCTTGGTCGCTTC | *ctrA* reverse primer for Cysteine mutation |
| CtrA C-term gsp 3 | ACTCTTCTTCGACTGGGGGCAATATGGAGCTGGTGCAGGCGCTGGAGCCG | *ctrA* forward primer for C-term mutation |
| CtrA C-term gsp 6 | ATATTGCCCCCAGTCGAAGAAGAGT | *ctrA* reverse primer for C-term mutation |
| HhoA -gsp5 | CTACAGTTGAGCCTATGCACAAG | *hhoA* 3´ ORF end |
| HhoA -gsp6 | GGCCTTCTTGTTCTTAGCAGTC | *hhoA* 3´ ORF end |
| HhoA -gsp6* | GACTGCTAAGAACAAGAAGGCCGGAGCTGGTGCAGGCGCTGGAGCC | pyrG*^Af^*/mrfp::pyrG*^Af^* cassette |
| HhoA -gsp3´ | CCAAGCCAGGCTGCCTGTCTGTCTGAGAGGAGGCACTGATGC | mrfp::pyrG*^Af^* cassette |
| HhoA -gsp3 | ACAGGCAGCCTGGCTTGG | *hhoA* 3´ UTR |
| HhoA -gsp4 | GCAACAGTCGACAGCACAGC | *hhoA* 3´ UTR |
